# Real-world experience of emicizumab prophylaxis in Korean children with severe hemophilia A without inhibitors

**DOI:** 10.1007/s44313-024-00039-1

**Published:** 2024-10-18

**Authors:** Sung Eun Kim, Ji Yoon Kim, Jeong A Park, Chuhl Joo Lyu, Seung Min Hahn, Jung Woo Han, Young Shil Park

**Affiliations:** 1 Department of Pediatrics, Kyungpook National University Hospital, Kyungpook National University Chilgok Hospital, 807 Hoguk-Ro, Buk-Gu, Daegu, 41404 Republic of Korea; 2 Department of Pediatrics, School of Medicine, Kyungpook National University, 680 Gukchaebosang-Ro, Jung-Gu, Daegu, 41944 Republic of Korea; 3 Department of Pediatrics, Inha University Hospital, 27 Inhang-Ro, Jung-Gu, Incheon, 22332 Republic of Korea; 4 Department of Pediatrics, Yonsei University Severance Children’s Hospital, 50-1 Yonseiro, Seodaemun-Gu, Seoul, 03722 Republic of Korea; 5 Department of Pediatrics, Kyung Hee University Hospital at Gangdong, 892 Dongnam-Ro, Gangdong-Gu, Seoul, 05278 Republic of Korea

**Keywords:** Emicizumab, Hemophilia A, Prophylaxis, Pediatric, Real-world data

## Abstract

**Purpose:**

Hemophilia A is a genetic disorder characterized by a lack of factor VIII (FVIII). Emicizumab, a recombinant humanized bispecific monoclonal antibody, mimics the function of FVIII. In this article, we present data on an initial real-world evaluation of emicizumab use in Korean children with severe hemophilia A without inhibitors.

**Methods:**

This study was conducted from June 2020 to March 2024 at 4 centers in Korea. The participants were pediatric patients with severe hemophilia A without inhibitors who had received emicizumab treatment for over 6 months. The mean and median annualized bleeding rates (ABRs) and mean and median annual joint bleeding rates (AJBRs) were compared.

**Results:**

Each of the 21 patients in the study received an emicizumab loading regimen of 3 mg/kg weekly for 4 weeks, followed by a modified maintenance regimen of which 2 patients (9.5%) received a 1.5 mg/kg weekly dose, 3 patients (14.3%) received a 6 mg/kg dose every 4 weeks, and the remaining 16 patients (76.2%) received a 3 mg/kg dose every 2 weeks. Before emicizumab prophylaxis initiation, the mean and median ABRs for all patients were 7.04 (SD ± 5.83) and 6.52 (range 0–21.74), respectively. After receiving emicizumab treatment, the mean and mediam ABRs decreased to 0.41 and zero, respectively. Additionally, 85.7% of the patients achieved no bleeding events within 6 months of starting the treatment.

**Conclusion:**

These first real-world data in Korea indicate that emicizumab is effective and safe for pediatric patients with severe hemophilia A without inhibitors.

## Introduction

Hemophilia A is a congenital disorder characterized by a deficiency of coagulation factor VIII (FVIII), leading to frequent bleeding. More than half of the patients with hemophilia A exhibit severe disease, defined as less than 1% of normal FVIII activity [1]. The standard therapy for severe hemophilia A is intravenous administration of FVIII concentrate products [2]. In the 1960s, the efficacy of prophylaxis with FVIII concentrate in preventing hemophilic arthropathy was investigated in several control trials, the results of which prompted many clinicians to advocate for the treatment [3–6]. Nevertheless, even with ongoing prophylactic treatment from childhood, hemophilic joint disease progresses owing to micro-bleeding in the joints during adulthood [3]. Moreover, prophylactic treatment requires the intravenous administration of FVIII concentrate 2 to 3 times a week, which burdens patients with frequent intravenous access and high costs. In particular, venous access is difficult in children, and some patients require implantable venous access devices [7].

Emicizumab, a recombinant humanized bispecific monoclonal antibody, mimics the activity of FVIII by bridging activated FIX and FX to generate activated FX [8]. Furthermore, because of its distinct antigenicity from FVIII, emicizumab is not expected to induce FVIII inhibitors [9]. Several clinical trials cited in the 3rd edition of the World Federation of Hemophilia guidelines have demonstrated that prophylactic treatment with emicizumab can significantly reduce the annual bleeding rate (ABR) in patients [10, 11]. In this research article, we present the first real-world analysis of emicizumab use in Korean pediatric patients with severe hemophilia A without inhibitors. Our study offers basic information on this specific subset of pediatric patients who used emicizumab, examines bleeding patterns before and after emicizumab use, and analyzes the serum drug concentration up to 24 weeks post-treatment, thereby providing insights into the conditions of Korean pediatric patients on this drug.

## Methods

### Data collection

This study, conducted from June 2020 to March 2024 across 4 centers in Korea, received institutional review board (IRB) approval from all institutions (Kyungpook University Hospital IRB; Approval Number: 2022–11-031) and was performed in accordance with the Declaration of Helsinki. Data collection began in December 2022. Given the retrospective nature of the study, consent and assent were waived. All participants were Korean pediatric patients with severe hemophilia A without inhibitors who had undergone emicizumab treatment for over 6 months at the time of study enrollment. Data on demographic characteristics, prior hemophilia A treatment regimens, emicizumab treatment regimens, bleeding patterns before and after emicizumab use (spanning from 6 months before to 6 months after therapy initiation), and serum emicizumab concentrations were extracted. The ABR was calculated as 365.25 times the number of bleeding events divided by the number of treatment days. The data cutoff date was March 30, 2024.

### Statistical analysis

Comparisons were made between the mean and median ABRs, the presence of target joints, the mean and median annual joint bleeding rates (AJBRs), and the proportion of patients experiencing no bleeding events before and after emicizumab administration. The categorical variables (i.e., characteristics of the study participants) were reported as counts and percentages, whereas the continuous variables were specified as means with standard deviation or medians with range. The Wilcoxon signed-rank test was used for comparing the ABRs before and after emicizumab treatment.

## Results

### Baseline characteristics (Table 1)

**Table 1:** Characteristics of the patients

	**Pediatric Patients Without Inhibitors**
Total number, N	21
Median age at the start of emicizumab (range)	4.00 (0–11)
Prior treatment regimen, N (%)	
Prophylaxis	20 (95.2)
On demand	1 (4.8)
Previous drug usage, N (%)	
pdFVIII	3 (14.3)
rFVIII	18 (85.7)
Presence of target joint, N (%)	3 (14.3)

The study included 21 pediatric patients, with a median age for starting emicizumab treatment of 4.0 years (range 0–11). Genetic data were obtained from only 7 patients, with 2 frameshift mutations, 1 missense mutation, 1 large deletion, and 3 intron 22 inversions identified. Three patients had a target joint before starting emicizumab. One patient was treated on demand with FVIII concentrate, whereas the other 20 patients were on prophylaxis therapy. Of the 21 patients, 3 received plasma-derived FVIII concentrate (pdFVIII) and 18 were administered recombinant FVIII concentrate (rFVIII).

### Results of emicizumab use

**Fig. 1:**
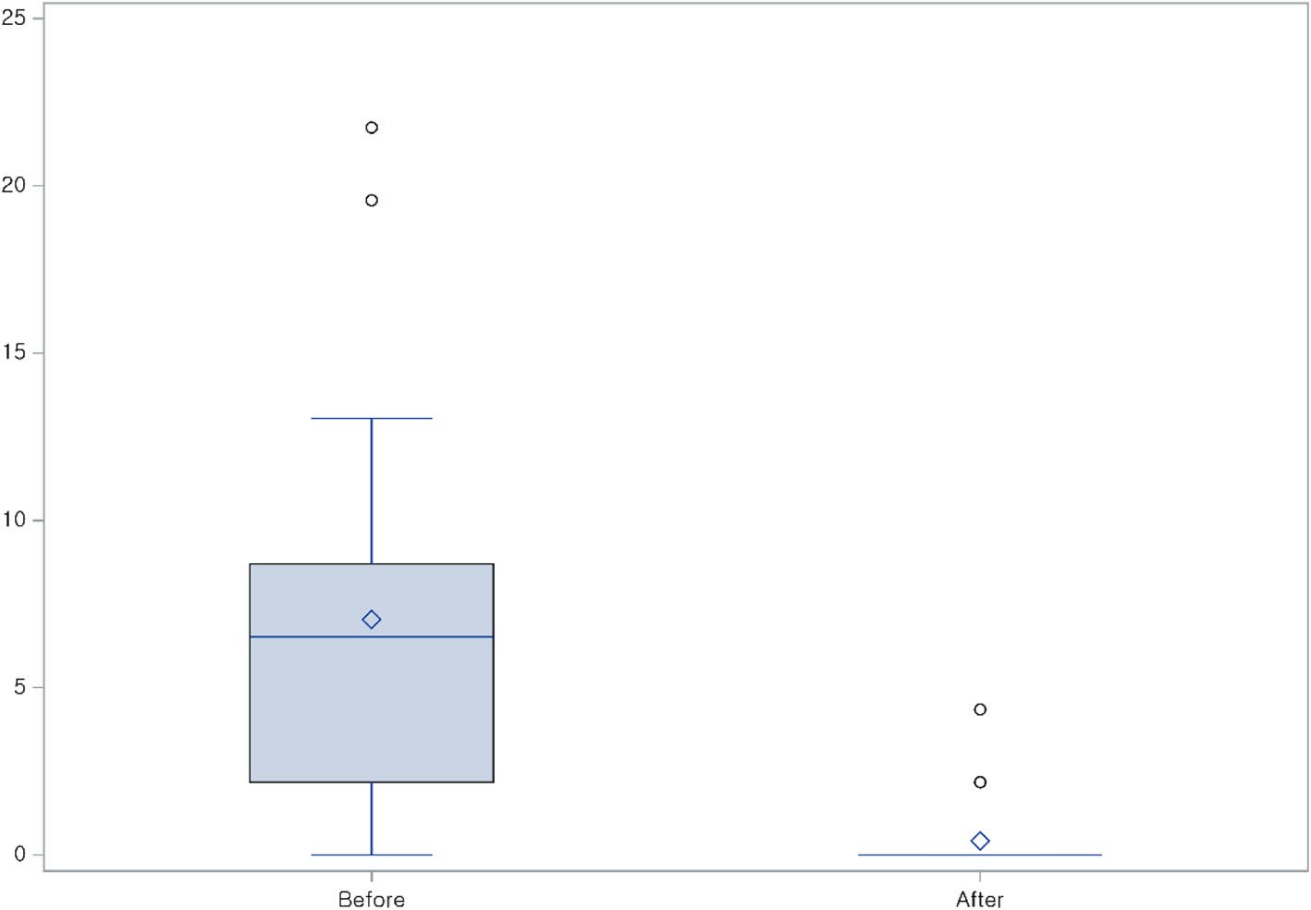
Comparison of pre-emicizumab and post-emicizumab annualized bleeding rates (*P* < 0.0001)

All patients underwent an emicizumab loading regimen (3 mg/kg weekly for 4 weeks), followed by a maintenance regimen modified to 1.5 mg/kg weekly for 2 patients (9.5%), 6 mg/kg every 4 weeks for 3 patients (14.3%), and 3 mg/kg every 2 weeks for the remaining 16 patients (76.2%) (Table 2). Prior to emicizumab initiation, the mean ABR for all the patients was 7.04 (SD ± 5.83), with a median ABR of 6.52 (range 0–21.74). Following emicizumab treatment, the mean and median ABRs decreased to 0.41 and zero, respectively (Fig. 1). Furthermore, 85.7% of the patients achieved no bleeding events within 6 months of initiating emicizumab (Table 3). Patient number 10 exhibited a post-emicizumab ABR of 4.35 with only 2 trauma-induced bleeding events during this period, which were resolved after a single administration of FVIII concentrate (Table 2).

**Table 2:** Details of individual patients

Patient Number	Age^a^ (Years)	Previous Drug Usage	Previous Treatment Method	Emicizumab Regimen	Pre-Emicizumab ABR	Post-Emicizumab ABR
1	0^b^	pdFVIII	On demand	Every 4 weeks	4.35	0
2	2	rFVIII	Prophylaxis	Every 4 weeks	8.7	0
3	1	rFVIII	Prophylaxis	Every 4 weeks	2.17	0
4	8	rFVIII	Prophylaxis	Every 2 weeks	2.17	2.17
5	9	rFVIII	Prophylaxis	Every 2 weeks	8.7	0
6	4	rFVIII	Prophylaxis	Every 2 weeks	2.17	0
7	2	rFVIII	Prophylaxis	Weekly	4.35	0
8	5	rFVIII	Prophylaxis	Every 2 weeks	2.17	0
9	10	rFVIII	Prophylaxis	Every 2 weeks	4.35	0
10	7	rFVIII	Prophylaxis	Every 2 weeks	6.52	4.35
11	11	rFVIII	Prophylaxis	Every 2 weeks	6.52	0
12	4	rFVIII	Prophylaxis	Every 2 weeks	6.52	0
13	10	pdFVIII	Prophylaxis	Weekly	6.52	0
14	4	rFVIII	Prophylaxis	Every 2 weeks	21.74	0
15	11	rFVIII	Prophylaxis	Every 2 weeks	0	0
16	0^c^	pdFVIII	Prophylaxis	Every 2 weeks	2.17	0
17	3	rFVIII	Prophylaxis	Every 2 weeks	2.17	0
18	4	rFVIII	Prophylaxis	Every 2 weeks	13.04	0
19	2	rFVIII	Prophylaxis	Every 2 weeks	19.57	0
20	5	rFVIII	Prophylaxis	Every 2 weeks	13.04	0
21	9	rFVIII	Prophylaxis	Every 2 weeks	10.87	2.17

^a^Age at the start of emicizumab

^b^Patient no. 1 age: 4 months

^c^Patient no. 16 age: 5 months

pdFVIII: plasma-derived factor VIII; rFVIII: recombinant factor VIII

**Table 3:** Bleeding data

	**Pre-Emicizumab**	**Post-Emicizumab**	***P*-value**
Mean ABR (± SD)	7.04 (± 5.83)	0.41 (± 1.11)	< 0.0001*
Median ABR (range)	6.52 (0–21.74)	0 (0–4.35)	
Mean AJBR (± SD)	2.28 (± 2.52)	0.21 (± 0.65)	0.0010*
Median AJBR (range)	2.17 (0–6.52)	0 (0–2.17)	
Zero bleeding, N (%)	1 (4.8)	18 (85.7)	< 0.0001^$^

^*^ Wilcoxon signed-rank test

^$^ McNemar’s test

*ABR *Estimated annualized bleeding rate, *SD *Standard deviation, *AJBR *Estimated annualized joint bleeding rate

**Fig. 2:**
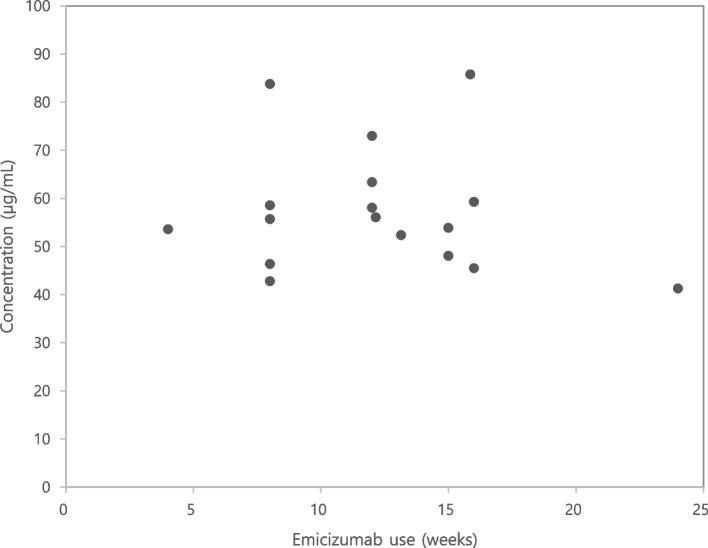
Emicizumab concentrations after treatment

There were no serious life-threatening adverse effects or events that led to the discontinuation or dose reduction of emicizumab. Additionally, no thromboembolic or systemic hypersensitivity reactions were reported. In 17 of the 21 patients, an emicizumab concentration test was performed within 24 weeks of treatment initiation, revealing a concentration range of 41.3–85.8 µg/mL (Fig. 2). All 19 patients tested with the chromogenic FVIII antibody assay had negative results.

In the following paragraphs, we describe two clinical cases of emicizumab-treated pediatric patients in the maintenance phase. One of them underwent an invasive procedure, and the other who had developed an intracranial hemorrhage was started on emicizumab at less than 1 year of age.

### Case 1

A 10-year-old male patient, previously on rFVIII prophylaxis, transitioned to emicizumab in May 2023 and initiated a maintenance regimen of 3 mg/kg biweekly in June 2023. His serum emicizumab concentration was 56.1 µg/mL at 12 weeks after treatment initiation, and he experienced no bleeding events. A chemoport removal operation was performed 4 months after emicizumab initiation, with presurgical administration of a single rFVIII concentrate dose ensuring successful bleeding control. He continues to receive maintenance therapy every 2 weeks without any subsequent bleeding events.

### Case 2

A 5-month-old boy was presented to the emergency room with a chief complaint of seizures. Although no clear history of trauma was evident, intracranial and subdural hemorrhages were revealed on brain computed tomography and magnetic resonance imaging scans. Additionally, during a blood test in the emergency room, hemostasis was poorly achieved, resulting in a hematoma at the venipuncture site on the left arm. The FVIII test value was 0.6%, leading to a diagnosis of severe hemophilia A, whereupon pdFVIII concentrate was administered to control bleeding. The patient had difficulty maintaining a peripheral venous catheter, prompting the planning of insertion of a chemoport to facilitate his frequent need for pdFVIII concentrate administration. However, his parents declined the chemoport insertion operation and the peripheral venous catheter was maintained, and the patient commenced emicizumab prophylaxis at 6 months of age. At the time of switching to emicizumab, the patient had been exposed to pdFVIII concentrate for 20 days, and an FVIII antibody test on the final day of pdFVIII concentrate administration was negative. He is currently on a maintenance emicizumab dose of 3 mg/kg once every 2 weeks and has experienced no bleeding events to date.

## Discussion

Unlike those for individuals with severe hemophilia A with inhibitors, studies on the long-term efficacy and safety of emicizumab for patients with severe hemophilia A without inhibitors have only recently been reported, with clinical trials such as HAVEN 3 and HOHOEMI having demonstrated positive outcomes. In HAVEN 3, a Phase 3 study assessing the safety and efficacy of emicizumab prophylaxis for ABR reduction, 152 patients with severe hemophilia A without inhibitors (age ≥ 12 years) were evaluated. The ABRs were 1.5 events for the once-weekly emicizumab prophylaxis cohort (Group A, *n* = 36) and 1.3 events for the once every 2 weeks cohort (Group B, *n* = 35). The ABR was 96% lower in Group A and 97% lower in Group B compared with that in the no prophylaxis cohort (Group C, *n* = 18). Furthermore, an intra-individual comparison of 48 patients who had been on FVIII prophylaxis and switched to once-weekly emicizumab prophylaxis showed a 68% reduction in ABR from that of the previous FVIII prophylaxis [12, 13]. A recent multicenter study in Japan assessed the safety and efficacy of emicizumab prophylaxis in pediatric patients with severe hemophilia A without inhibitors (age < 12 years) [12]. Among these participants, one was a previously untreated patient and 12 had been on FVIII prophylaxis. The ABRs were 1.3 events for the once every 2 weeks cohort (*n* = 6) and 0.7 events for the once every 4 weeks cohort (*n* = 7) [12].

Although the scale of our study of Korean children was small, it demonstrated positive results. After emicizumab was marketed in Korea in January 2019 and patients with hemophilia A with inhibitors began receiving national insurance reimbursement for the drug in May 2020, a multicenter trial was conducted to confirm the safety and effectiveness of this prophylactic treatment [14]. In Korea, patients with hemophilia A without inhibitors have been eligible for national insurance reimbursement for emicizumab since May 2023. This first real-world study of emicizumab prophylaxis for pediatric patients with severe hemophilia A without inhibitors showed that all patients achieved ABR reduction. In real-world settings, the maintenance doses are 1.5 mg/kg once weekly, 3 mg/kg once every 2 weeks, or 6 mg/kg once every 4 weeks depending on patient convenience and preference. In this study, the preferred maintenance dose regimen for most of the patients (76.2%) was 3 mg/kg once every 2 weeks. Regardless of the chosen regimen, bleeding was significantly reduced after the use of emicizumab for 24 weeks.

In the HOHOEMI trial, plasma emicizumab concentrations were compared among pediatric patients with severe hemophilia A without inhibitors who were administered the drug every 2 or 4 weeks. Upon completion of the loading dose, the average concentrations were 48.7 and 48.4 µg/mL in the 2- and 4-weekly cohorts, respectively. Additionally, the mean steady-state trough concentrations for the two cohorts were 35 µg/mL (range 20.9–50.5 µg/mL) and 30 µg/mL (range 13.4–55.2 µg/mL), respectively [12]. In the HAVEN 3 trial, emicizumab concentrations were compared in patients administered the drug every week or 2 weeks, with trough concentrations being effectively maintained in both cohorts [13]. Emicizumab concentrations were measured once per patient over 24 weeks. At a steady state, an emicizumab trough concentration of 30 µg/mL or higher is considered sufficient to achieve almost the maximum effect of the drug [15], indicating that all trough concentrations measured in our study were appropriate. In both cohorts of the HOHOEMI study, the ABRs for treated bleeding events were low and showed no clear difference, even when compared with the ABRs from the HAVEN 3 and HAVEN 4 trials (the latter in which emicizumab was administered every 4 weeks to patients ≥ 12 years of age with severe hemophilia A, with or without inhibitors), despite slightly different mean steady-state trough concentrations. The absence of a further reduction in ABR with higher trough concentrations aligns with recent quantitative analyses showing that the relationship between plasma emicizumab concentrations and ABR almost reaches a plateau at concentrations above approximately 30 μg/mL. This is similar to the reported relationship between FVIII activity and ABRs that suggests a minimized risk of joint bleeding at concentrations above 10 IU/dL [12].

However, asynchronous progression and the variation in emicizumab concentrations across patients are limitations to the prophylactic treatment. With national insurance reimbursement for emicizumab now available to patients with hemophilia A without inhibitors, a higher number of patients with non-antibody severe hemophilia A are expected to start using the drug and more data will be collected. As more patients opt for different maintenance regimens and long-term use of emicizumab, data on plasma emicizumab concentrations will accumulate and the correlation between maintenance intervals, emicizumab concentrations, and ABRs can be established.

In conclusion, this study yielded the first real-world data on emicizumab prophylaxis in Korean pediatric patients with severe hemophilia A without inhibitors. The findings indicate the significant efficacy of the drug in stably reducing the ABR without serious adverse events.

## Data Availability

The datasets generated and/or analyzed during the current study are available from the corresponding author on reasonable request. The data is stored in a secure institutional repository and is not publicly accessible due to privacy restrictions.
